# Decrypting orphan GPCR drug discovery via multitask learning

**DOI:** 10.1186/s13321-024-00806-3

**Published:** 2024-01-23

**Authors:** Wei-Cheng Huang, Wei-Ting Lin, Ming-Shiu Hung, Jinq-Chyi Lee, Chun-Wei Tung

**Affiliations:** Institute of Biotechnology and Pharmaceutical Research, National Health Research Institutes, Miaoli County, 35053 Taiwan

**Keywords:** Multitask learning, G protein-coupled receptors, GPCR, Feature selection, Ligand-based virtual screening

## Abstract

The drug discovery of G protein-coupled receptors (GPCRs) superfamily using computational models is often limited by the availability of protein three-dimensional (3D) structures and chemicals with experimentally measured bioactivities. Orphan GPCRs without known ligands further complicate the process. To enable drug discovery for human orphan GPCRs, multitask models were proposed for predicting half maximal effective concentrations (EC_50_) of the pairs of chemicals and GPCRs. Protein multiple sequence alignment features, and physicochemical properties and fingerprints of chemicals were utilized to encode the protein and chemical information, respectively. The protein features enabled the transfer of data-rich GPCRs to orphan receptors and the transferability based on the similarity of protein features. The final model was trained using both agonist and antagonist data from 200 GPCRs and showed an excellent mean squared error (MSE) of 0.24 in the validation dataset. An independent test using the orphan dataset consisting of 16 receptors associated with less than 8 bioactivities showed a reasonably good MSE of 1.51 that can be further improved to 0.53 by considering the transferability based on protein features. The informative features were identified and mapped to corresponding 3D structures to gain insights into the mechanism of GPCR-ligand interactions across the GPCR family. The proposed method provides a novel perspective on learning ligand bioactivity within the diverse human GPCR superfamily and can potentially accelerate the discovery of therapeutic agents for orphan GPCRs.

**Supplementary Information:**

The online version contains supplementary material available at 10.1186/s13321-024-00806-3.

## Introduction

In eukaryotes, the G protein-coupled receptors (GPCRs) superfamily is one of the largest and most diverse families of transmembrane receptor proteins. The heterotrimeric G proteins composed of Gα, Gβ, and Gγ subunits interact with the C-terminus of GPCRs to stimulate many signaling functions [1]. When GPCRs are activated, Gα dissociates from Gβ and Gγ, allowing the two subunits to exert their respective downstream signaling roles. While GPCRs have been recognized as the cellular membrane receptors for multiple ligands such as biological amines, amino acids, ions, lipids, peptides/proteins, light, odorants, pheromones, nucleotides, and opiates, the precise roles and pathways of GPCRs as receptors for animal steroid hormones, including those of insects, remain incompletely determined [2]. The human genome has identified more than 800 GPCRs, which can produce various biological responses through specific ligand interactions [3]. The human GPCRs are divided into classes based on sequence homology and functional similarity using the GRAFS system acronym (Glutamate, Rhodopsin, Adhesion, Frizzled/Taste2, Secretin); that is for class A (Rhodopsin receptors), class B (in that two subfamilies: secretin receptors (B1) and adhesion receptors (B2)), class C (metabotropic Glutamate receptors), class F (Frizzled/smoothened receptors), and class T (taste 2 receptors) [4, 5]. Despite the lack of sequence homology between classes and the high variability in length of GPCRs, all GPCRs share a typical barrel-shaped architecture with seven transmembrane α-helices, which consist of three intracellular loops and three extracellular loops, and the C-terminus intracellular for the interaction of downstream effectors. This barrel-shaped structure forms a cavity in the plasma membrane, and functions as the ligand-binding region of the receptor, and large ligands, such as proteins and peptides, may also bind to extracellular loops [6, 7].

Among currently available drugs, GPCRs are important drug targets, accounting for approximately 35% of all drugs approved by the Food and Drug Administration (FDA) against this membrane protein family [7]. In particular, it was estimated that about half of the current market drug targets are GPCRs, mainly because of their involvement in signaling pathways related to many diseases, such as psychiatry, metabolism (including endocrine diseases), immunology (including viral infection), cardiovascular, inflammation, sensory disturbances, and cancer. There are more than 200 human GPCRs identified with their physiological ligands. Still, about 120 GPCRs have not yet been identified as endogenous ligands. These so-called orphan GPCRs represent an unexplored area of GPCR drug discovery [8]. In new drug development, compound-protein interaction is the main method used. Recent deep-learning non-homology-based structural prediction tools were utilized in many cases with promising results, such as AlphaFold2 and RoseTTAFold [9–11]. However, the average root-mean-square deviation of atomic positions (RMSD) of the predicted target GPCR protein structures from the neural network-based methods against known structures were significantly different, with RMSD greater than 5 angstroms for both predictors [10]. While traditional protein–ligand docking algorithms provide powerful tools for identifying ligands, they were even hampered without the known structure of the less than 50% protein sequence similarity for orphan GPCR proteins. Consequently, traditional compound-protein interaction methods are unsuitable for drug discovery of orphan G proteins.

**Fig. 1:**
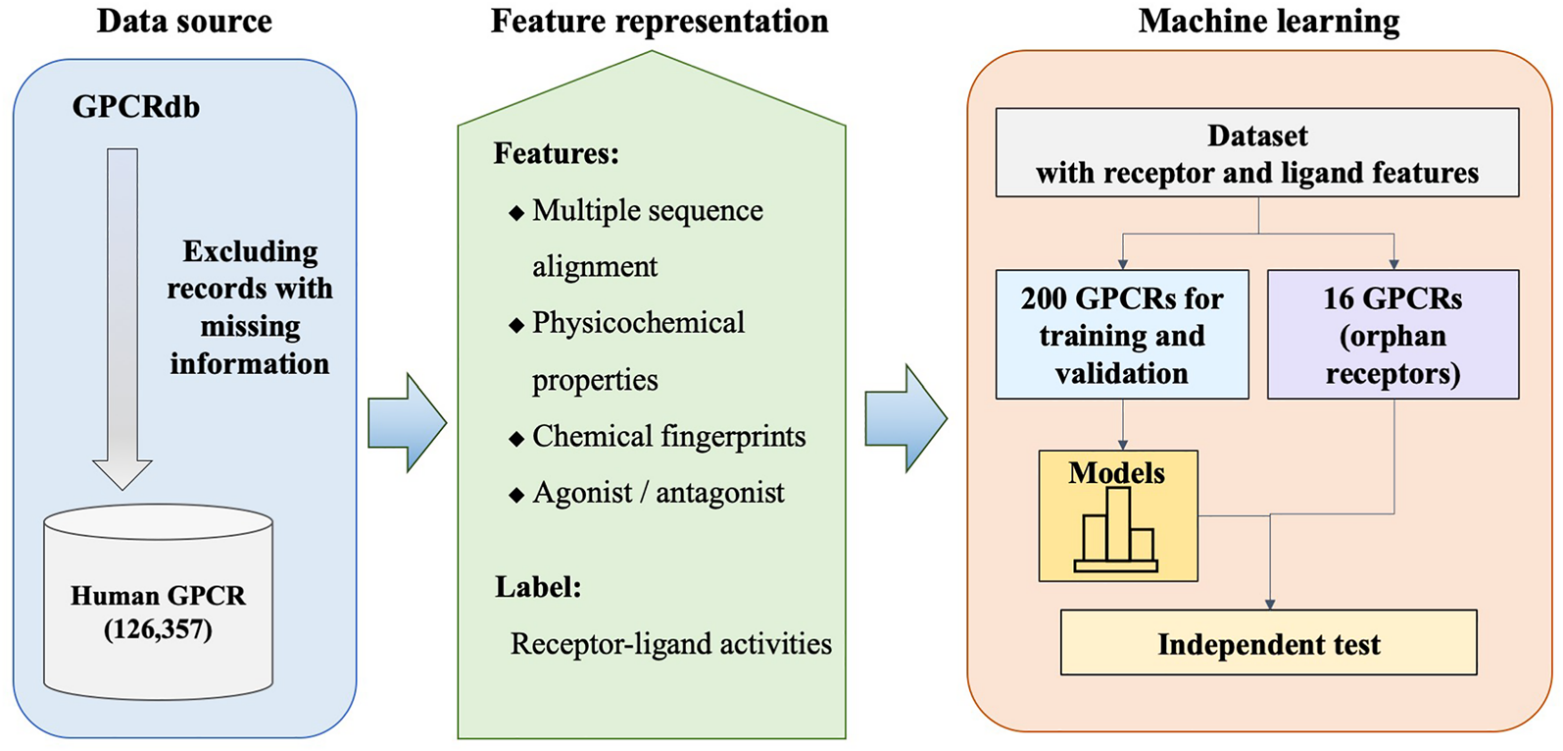
Flowchart of the stacked ensemble multitask learning models for the GPCR bioactivities. The human GPCR-ligand pair activities database was extracted from GPCRdb. The models were generated from the individual or integrated receptors training datasets. The validation datasets and test orphan datasets validated the models independently

Machine learning models for GPCR have been developed rapidly in three streams. One direction was to discriminate between agonists and antagonists for GPCRs [12]; however, some ligands were found to play partial agonist and partial antagonist roles, which do not induce a 100% full activation state [13]. Consequentially, another direction was regression models of GPCR-ligand pair activity by using ligand-based and structural docking-based machine learning algorithms [14–16], and the stereo-based training methods using reported protein data bank (PDB) structures and molecular structures, such as pdCSM-GPCR [17], and neural network models using voxelization of GPCR and ligand structures [15]. The third direction was the protein–protein interaction models of higher-order GPCR molecular complexes with the other GPCR protein pairs [18, 19]. The development of orphan GPCR-targeted drugs is challenging due to the complex and diverse nature of the GPCR family. Because of the absence of protein–ligand activities for the orphan GPCRs, they were limited using structure-based approaches [15, 20]. Considering some conserved motifs observed from previous GPCR-ligand interaction studies [21], it is therefore interesting to identify interaction patterns from existing data and transfer the knowledge of these patterns for ligand recognition of orphan GPCRs. This study presents a novel method for developing multitask models to predict GPCR-ligand activities of orphan receptors using features of protein sequence, physicochemical properties, and chemical fingerprints. The proposed method utilizing multitask learning to extract common ligand recognition patterns from known ligand-target pairs showed promising performance for predicting half maximal effective concentration (EC_50_) of ligands for validation and test orphan datasets with MSE of 0.24 and 1.51, respectively. By integrating protein and chemical features, the developed prediction model offers a novel approach to decrypt the hidden messages of pair bioactivities between ligand and orphan GPCRs. In addition to prediction models, the protein features were analyzed, and the N-terminal region showed outstanding significance, providing insights into the mechanism of GPCR-ligand recognition beyond the structural knowledge. The identified residues and chemical properties provided a deeper understanding of the mechanisms underlying GPCR-ligand interactions for discovering therapeutics targeting GPCRs (Fig. 1).

## Methods/experimental

Programs were developed in the Ubuntu 20.04.3 operating system using Python programming language version 3.7.11. The study used several Python packages, including numpy, pandas, matplotlib, beautifulsoup4, scikit-learn, bitarray, rdkit-pypi, torch, and AutoGluon v0.5.2. These packages were used for various tasks of data manipulation, visualization, machine learning, web scraping, and deep learning, respectively.

### Datasets

GPCRdb database [22] includes bioactivity information from ChEMBL [23] on multiple-species GPCRs and their paired ligands. As of 2021, it contains 471,355 GPCR-ligand pair bioactivities (Access date: 2021.11), which includes 369,843 human GPCR-ligand pair bioactivities. In this study, we consider only human GPCR-ligand interactions. By excluding the records without information on agonist, antagonist, and EC_50_, the remaining 126,357 records of 66,165 GPCR-agonist and 60,192 GPCR-antagonist pair bioactivities of EC_50_ values in nanomolar were utilized in this study. The dataset comprised 216 unique human GPCR receptors and 49,022 unique chemical ligands. Among the 216 GPCRs, 181 GPCRs are associated with agonist data, and 177 GPCRs are associated with antagonist data. In order to simulate the prediction on orphan GPCRs, 16 GPCR receptors containing less than 8 bioactivities were employed as orphan GPCR datasets for further testing of the models. The remaining 200 GPCRs were utilized for model training and validation. For each GPCR and agonist/antagonist activity, the corresponding EC_50_ records were split into a training set and a validation set with a ratio of 0.8 and 0.2. The training sets and validation sets were then merged into a final training dataset and validation dataset, respectively, for the multitask model development.

### Feature encoding

For the multitask learning, each GPCR-ligand pair data was encoded as a 5023-dimensional feature vector consisting of GPCR protein sequence alignment, physicochemical properties, and fingerprints of paired ligands. The GPCR protein sequences were obtained from the Universal Protein Resource (UniProt) [24] and aligned using MUSCLE 3.8.31 [25]. The gap positions in the alignment sequence were padded with dashes, and the multiple sequence alignment results were further encoded according to amino acid properties. That is 0 for padding; 1 for amino acid with special side chains, C, G, P, and A; 2 for amino acid with hydrophobic side chains, V, I, L, M, F, Y, and W; 3 for amino acid with polar uncharged side chains, S, T, N, and Q; and 4 for amino acid with electrically charged side chains, D, E, R, H, and K. A 2,554-dimensional vector was obtained for each GPCR protein. The simplified molecular input line entry specification (SMILES) [26] representing ligand structures were obtained from GPCRdb and encoded using PaDEL-descriptor to calculate 1,444 features of physicochemical features [27] and using RDkit to calculate 1,024 binary representations of extended-connectivity fingerprints with a maximum diameter of 6 atoms (ECFP) [28, 29] and one binary feature indicating an agonist and antagonist interaction. The logarithm of the corresponding EC_50_ activities was used as a label for the models' development.

### Multitask model development

For comparison, the single-task learning models for GPCRs and the multitask learning models were implemented. The single-task learning models for agonist activity of individual GPCRs (STL-AG) and single-task learning models for antagonist activity of individual GPCRs (STL-ATG) were developed and validated using the corresponding training and validation sets. For the multitask learning model, the training and validation sets were merged and utilized to develop the multitask models for agonist activity (MTL-AG), antagonist activity (MTL-ATG), and a merged of agonist and antagonist activity (MTL-AG-ATG).

Five algorithms were utilized to develop prediction models, which include neural networks, LightGBM boosted trees [30], CatBoost boosted trees [31], random forests [32], and extremely randomized trees [33]. To improve the training performance, the models trained based on these algorithms were further ensembled and stacked [34] using the AutoGluon-Tabular framework [35], with a maximum 7-h training time. The mean squared error (MSE) was utilized as the objective function for model training. The MSE is calculated as the following *Eq. *1,

1 $$MSE=\frac{\sum_{i=1}^{n}{({Y}_{i}-{y}_{i})}^{2}}{n}$$

where ${Y}_{i}$ and ${y}_{i}$ are the experimental and predicted values of the instance. The *i* and *n* are the number of instances.

For performance evaluation, MSE and Pearson's correlation coefficient (CC) were utilized. The equation of CC was given in the following *Eq. *2,

2 $$CC=\frac{\sum \left({Y}_{i}-\overline{Y}\right)\left({y}_{i}-\overline{y}\right)}{\sqrt{\sum {\left({Y}_{i}-\overline{Y}\right)}^{2}\cdot \sum {\left({y}_{i}-\overline{y}\right)}^{2}}}$$

where $\overline{Y}$ is the mean of the experimental values of the variable being predicted, and $\overline{y}$ is the mean of the predicted values. The *i* is the number of instances.

### Feature selection

To identify the most relevant features for predicting GPCR-ligand pair activities, the minimum redundancy-maximum relevance (mRMR) feature selection algorithm [36] was utilized to identify top-ranked *m* features from datasets. The model training datasets were refined by selecting the most relevant protein and chemical property features in combination with the 1024 bits of ECFP and one binary agonist and antagonist. The datasets with selected features were divided into training and validation datasets with a ratio of 8:2. The training time was restricted by implementing a linear increase in the number of features, which was multiplied by 5 s, with a maximum limit of 7 h. The optimization model for feature selection was selected with less than a 5% significant improvement in the validation performance of MSE.

### Protein sequence similarity

The pairwise protein sequence similarities were calculated using the Tanimoto similarity. The Tanimoto similarity used the ratio of the number of intersecting sets to the number of union sets as the similarity measurements, excluding the intersecting missing sequence positions for the numerator (Eq. 3). X and Y are the aligned protein sequence features.

3 $$Tanimoto\, similarity \left(X,Y\right)=\frac{\left(X\bigcap Y\right)}{\left(X+Y-X\bigcap Y\right)}$$

### Statistical analysis

The statistical differences between the models were analyzed with the Mann–Whitney *U* test by Prism (GraphPad Software Inc., USA). A *p*-value < 0.05 was recognized as statistical significance.

## Results and discussion

### Model development

To develop prediction models for orphan GPCRs, a large dataset of 66,165 agonist and 60,192 antagonist activities (EC_50_) for 216 GPCRs was extracted from GPCRdb. The development of prediction models considered only 200 GPCRs with more than or equal to 8 activities. The other 16 GPCRs were utilized as orphan GPCR datasets to simulate the performance of discovering ligands for orphan GPCRs. Each sample was encoded as a high-dimensional feature vector consisting of multiple sequence alignments of GPCR, and physicochemical properties and fingerprints of the corresponding chemical. Datasets were randomly split into training and validation datasets in a ratio of 8:2, respectively.

**Fig. 2:**
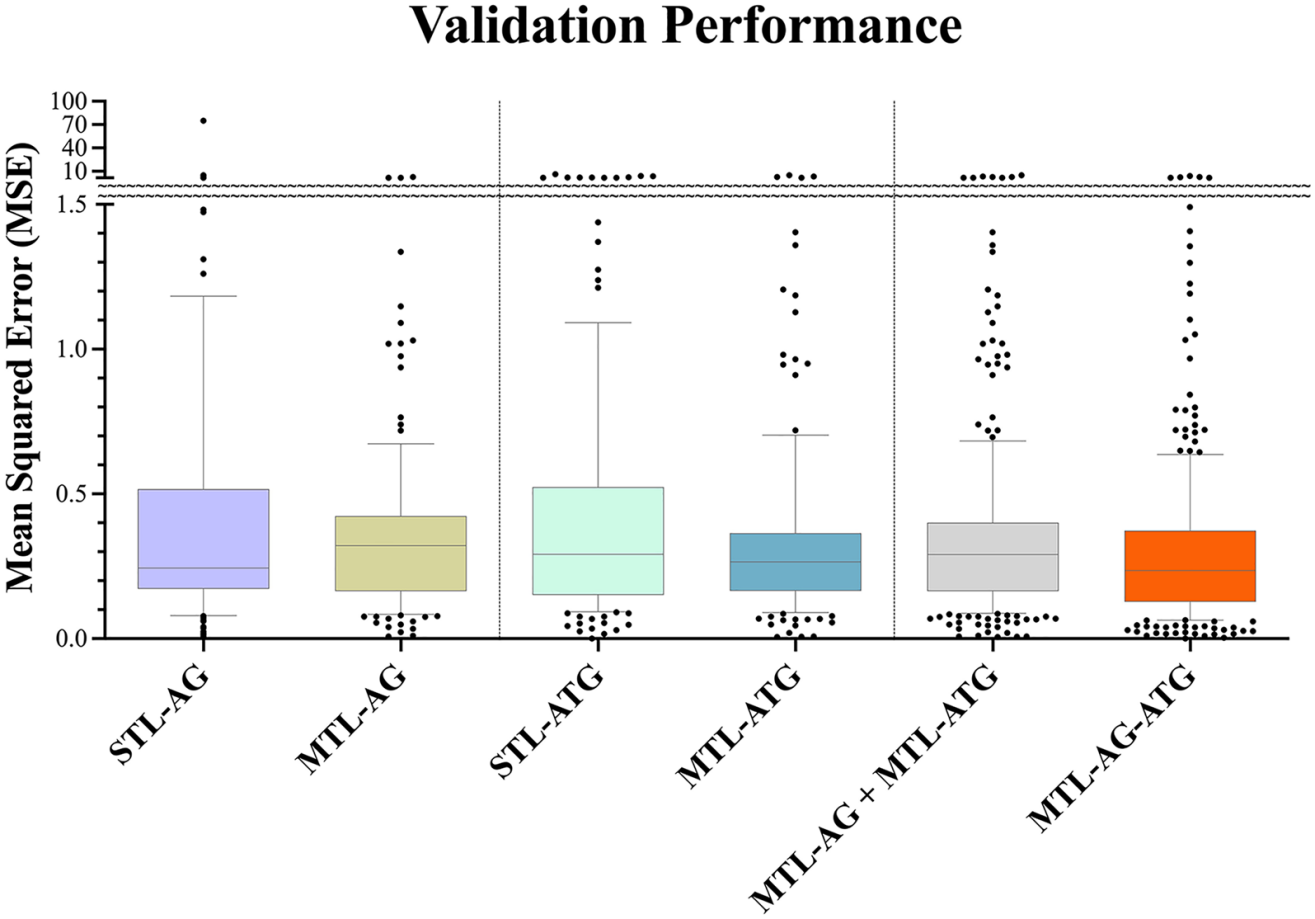
The box plotting of validation performance for the single task models and multitask models. STL-AG and STL-ATG presented the performances of single-task models trained using the individual receptor with agonist or antagonist datasets; MTL-AG and MTL-ATG for the performances of the multitask models trained using integrated receptors with agonist or antagonist datasets, the MTL-AG + MTL-ATG represented to the merged validation results of both models, and the MTL-AG-ATG for the integrated receptors with a merged of agonist and antagonist model using agonist datasets or antagonist datasets validation. The y-axis is the validation performance of the mean MSE for each GPCR

The multitask and single-task learning models were developed using five algorithms and their ensembles based on the AutoGluon-Tabular framework. Comparison of validation performances for the multitask and single-task models were shown in Fig. 2. The multitask model for agonists (MTL-AG) outperformed corresponding single-task models (STL-AG) with a 3.3-fold improvement on MSE, for which there were fewer data located beyond 1.5 of MSE (Fig. 2). Similarly, the multitask model for antagonists (MTL-ATG) exhibited superior performance over corresponding single-task models (STL-ATG) with a 1.85-fold improvement on MSE. Both the MTL-AG and MTL-ATG significantly improved performance in comparison to the single-task models of STL-AG and STL-ATG (p < 0.05), respectively. The MSE and CC values are 0.29 and 0.80 for MTL-AG, and 0.27 and 0.83 for MTL-ATG, respectively. The multitask model based on all agonist and antagonist data for 200 GPCRs (MTL-AG-ATG) presented an MSE of 0.24 and a CC of 0.85, of which the merged agonist and antagonist model presented a better performance than MTL-AG or MTL-ATG. The merged multitask model of MTL-AG-ATG not only improved performance by 10–20% of MSE but also spontaneously integrated receptors and merged ligand types provided a versatile and innovative approach to investigating the mechanisms of agonistic and antagonistic ligand interactions. Please refer to Additional file 1: Table S1 for detailed performance measurements.

To explore the possibilities of enhancing the multitask model, the chemical features were replaced by a 300-dimensional vector generated from a pre-trained model of Mol2vec [37]. The resulting models of MTL-AG-ATG-M2V showed an average MSE of 0.27 and an average CC of 0.85, representing an 11% performance decrease in MSE when compared to the MTL-AG-ATG model (Additional file 1: Table S1). The same performance decrease was obtained for MTL-AG-ATG-M2V-FS using Mol2vec.

### Protein feature truncation and permutation test

**Fig. 3:**
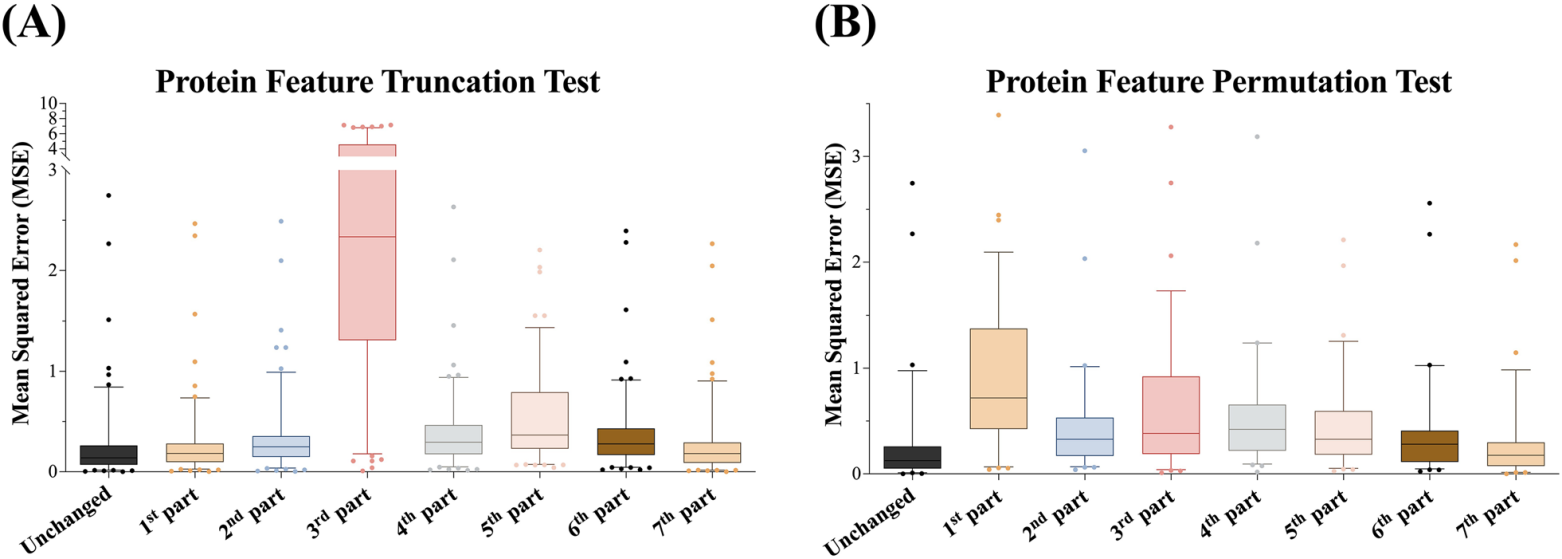
Truncation and permutation test for determining the informative protein alignment features. **A** Boxplot of the protein feature truncation test for the MTL-AG-ATG model. **B** Boxplot of the protein feature permutation test for the MTL-AG-ATG model

To reveal the mechanisms underlying agonistic and antagonistic ligand interactions in the multitask models of the integrated receptors with a merged agonist and antagonist (MTL-AG-ATG), 49 individual GPCR datasets with their validation performance of CC (correlation coefficient) greater than 0.95 against the MTL-AG-ATG model were selected for the tests. Considering the nature of the seven transmembrane helixes of GPCR, the full-length protein sequences of these datasets were divided equally into seven parts. Each part of the protein-coding was replaced with 0 for the truncation test datasets. The individual truncated datasets were tested against the multitask MTL-AG-ATG model. The test results showed that the higher error represented a higher impact of the protein sequence on the protein–ligand pair bioactivities. In other words, some of the protein sequences were must-have features. According to the test result, the middle parts of the GPCR protein were having a high impact on the truncation test, especially the 3rd part truncation test showed a significantly high error (Fig. 3A).

To further investigate the contribution of the protein parts to the EC_50_ prediction, seven permuted datasets obtained by applying randomized encoding for each of the seven parts of the protein sequence were utilized for the following permutation test. The permuted part with the largest performance decrement possesses the highest information of the protein–ligand pair's bioactivities. The permutation of 1st and 3rd parts of the protein features gave the highest perturbation of the MSE performance, where the MSE values were 0.9 and 0.7, respectively. Compared to the original MSE value of 0.19 for the selected 49 datasets, the MSE errors were perturbed by 4.5 and 3.5 folds, respectively. It suggested that the 1st and 3rd parts of the protein sequence features were more sensitive than the other parts, *i.e.*, the 2nd, 4th, and 5th parts. The results of the protein feature permutation test indicated that the N-terminal part of the protein has a greater impact on the model's performance compared to the other parts (Fig. 3B).

The truncation test indicated the ‘must have’ region of the protein–ligand interaction, which was observed with much GPCR-ligand structural biology evidence [38–41]. However, beyond structural information, the function of N-terminal GPCR remains unsettled with its ligand bioactivities. Consequently, the permutation test results highlighted the importance of N-terminal regions in GPCR proteins and introduced the possibility of optimizing the multitask model using a feature selection algorithm, in which some parts of GPCR protein sequences could be more important for predicting EC_50_ of paired ligands.

### Feature selection for the multitask model

**Fig. 4:**
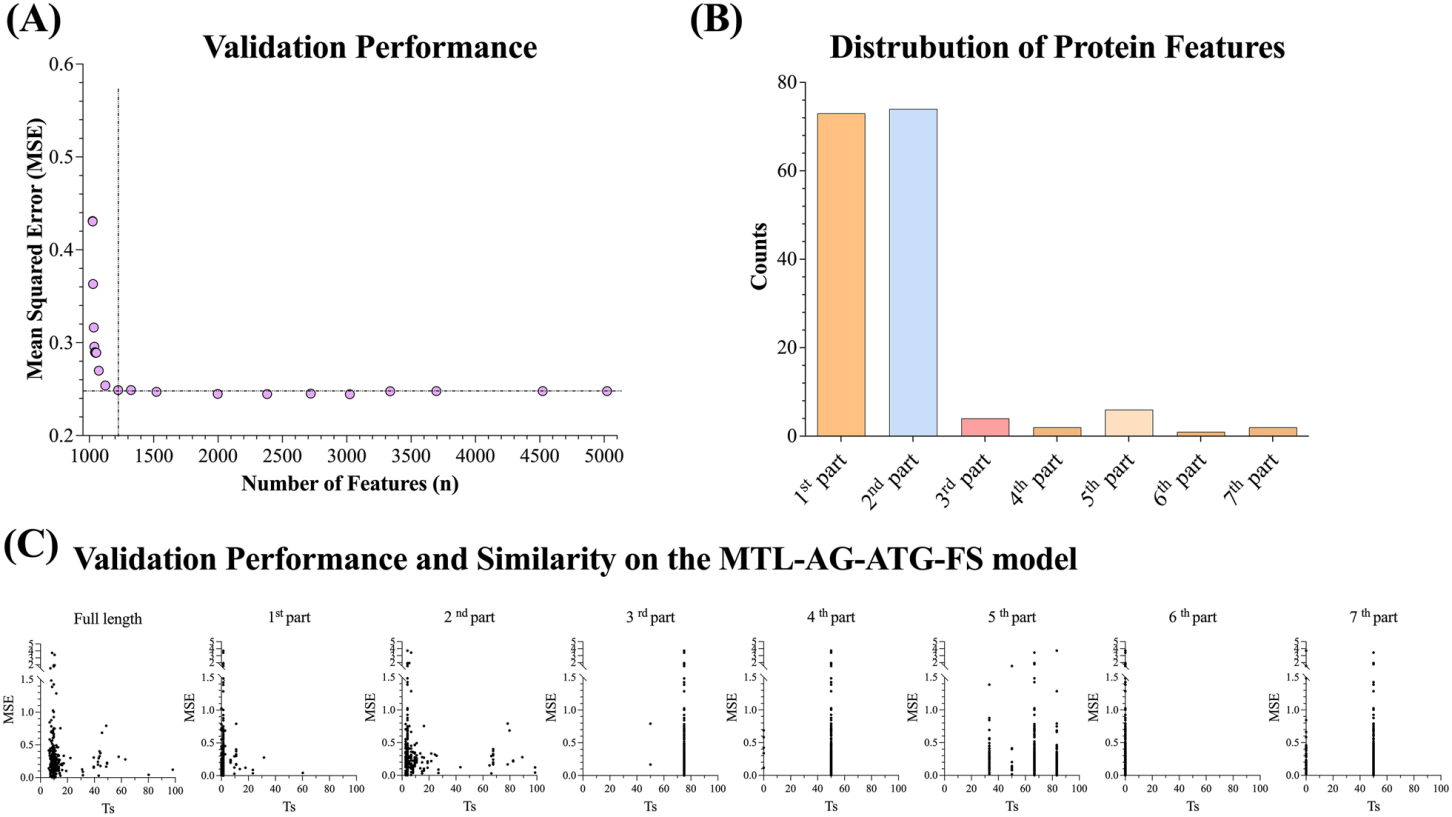
The mRMR-based feature selection. **A** The validation performance for top-ranked feature selection model sets. **B** Distribution analysis of 162 aligned protein features of top-ranked 200 features from the feature-selected model (MTL-AG-ATG-FS). **C** The scatter plot of pairwise Tanimoto similarity (*Ts*) based on selected protein features calculated as individual parts or full-length sequence plotting against validation performance on the MTL-AG-ATG-FS model

The minimum redundancy and maximum relevance (mRMR) algorithm was applied to select important features and optimize the model of MTL-AG-ATG. The multitask model with the top-ranked 200 features (MTL-AG-ATG-FS) was selected where the inclusion of additional features gave no significant improvement on the MSE performance (Fig. 4A; please refer to Additional file 1: Table S2). Please note that ECFP features do not undergo the feature selection process, the number of features in Fig. 4A represents the summation of 1,024 ECFP features and mRMR-selected features. A total of 1,224 features consisting of 162 protein alignment features, 38 physicochemical features, and 1,024 ECFP features were utilized for the following analysis (Additional file 1: Table S3). The feature selection approach was beneficial in terms of enhancement of model performance and reduction of the model training time (Additional file 1: Table S2). The MTL-AG-ATG-FS model integrated receptors and merged agonistic and antagonistic ligands showed an average MSE of 0.24, and an average CC of 0.85 with a quarter of the training time of 102 min than the all-feature multitask model of 420 min (MTL-AG-ATG) (Additional file 1: Table S1).

The majority of the selected protein features were located in the N-terminal region of the protein sequence (Fig. 4B). It is noteworthy that the results are consistent with previous studies that the N-terminus of the GPCR protein has been widely acknowledged for its importance in receptor translation and trafficking [42]. Moreover, it also aligns with the findings from the protein feature permutation test.

Since a protein target dissimilar to the training dataset may have different properties, it is important to investigate the relationship between the similarity of protein features and model performance. First, a pairwise similarity matrix for the GPCR protein features was calculated using Tanimoto similarity (*Eq. *3) based on the top-ranked 200 features. Subsequently, the maximum Tanimoto similarity (*Ts*) was calculated for each of the GPCRs. The *Ts* and the corresponding performance were shown in Fig. 4C. A lower MSE was observed for highly similar protein features in the 1st part, 2nd part, and the full length of aligned protein sequences. When applying the model to orphan receptors with dissimilar protein features, the *Ts* similarity may provide an important clue to the reliability of prediction.

### Application domain using protein sequence similarity

**Fig. 5:**
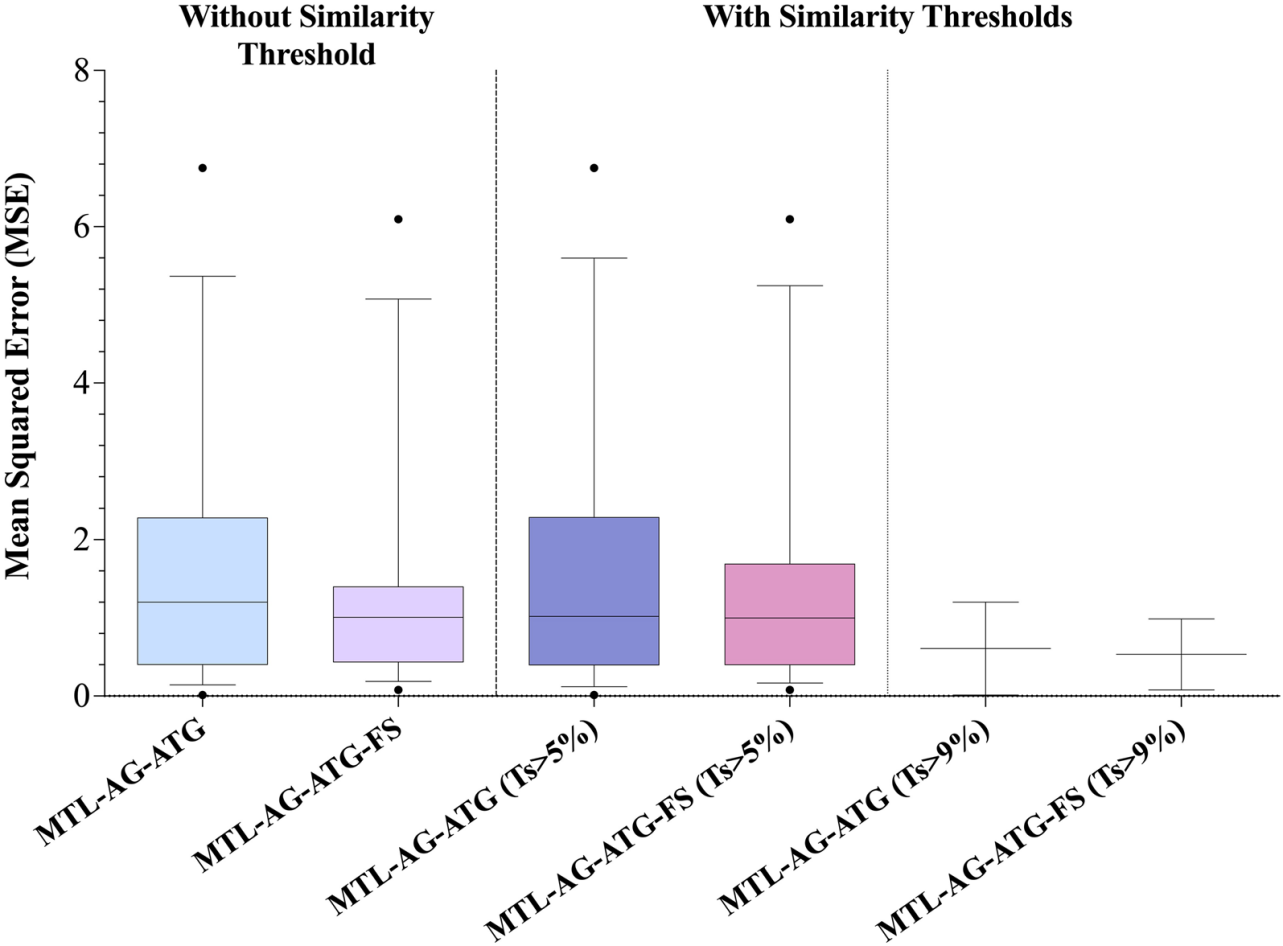
The independent test results on the orphan datasets of the multitask models with and without the similarity thresholds

Although the GPCRs belong to the same superfamily and share similar structural scaffolds, the similarities among protein sequences can be highly divergent. Since the transferability of the developed multitask model is largely based on protein sequence alignment, the investigation of the applicability domain of the model is therefore of interest. To provide better insights into the transferability of shared knowledge to orphan receptors, for each GPCR, its maximum Tanimoto similarity (*Ts*) to the other GPCRs was first determined using the top-ranked 200 features. By excluding the GPCRs with a *Ts* less than or equal to a specific threshold, the performances of the models on the orphan datasets were less divergent, and better overall performance were obtained (Figure S1). The feature-selected model (MTL-AG-ATG-FS) achieved a better MSE performance of 1.5 compared to the all-feature model (MTL-AG-ATG), which yielded an MSE of 1.7, for the orphan datasets (Additional file 1: Table S1). When a similarity threshold of 5% was applied to the independent test dataset, the performances to the independent test datasets of both models remained 1.5 and 1.7 for the MTL-AG-ATG-FS model and MTL-AG-ATG model, respectively (Fig. 5). Moreover, when the feature-selected model was subjected to a stricter similarity threshold of 9%, it demonstrated improved performance with an MSE of 0.5, whereas the all-feature model yielded an MSE of 0.6.

During the review process of the manuscript, two agonistic ligands of 7-fluorotryptamine and tryptamine were reported for the orphan GPRC5A [43]. With a Ts of 6.2%, a good MSE of 1.46 was obtained using the MTL-AG-ATG-FS model. Detailed prediction is listed in Additional file 1: Table S5. For the promising agonist of 7-fluorotryptamine, its predicted EC_50_ is 2.4 μM, which is close to the experimental value of 7.2 μM [43]. This provides a successful example for predicting ligands of an orphan receptor.

### Interpretation of the top-ranked protein features at the ligand–protein interaction level

The permutation test (Fig. 3A) and the distribution of the top-ranked protein features of the MTL-AG-ATG-FS model (Fig. 4B) coherently revealed the importance of the N-terminal region of the GPCRs. In order to correlate the relationship of protein structures and bioactivities of the GPCR, the 162 selected protein features from the feature-selected model (MTL-AG-ATG-FS) were highlighted in four selected structures representing four major classes of the GPCRs (A, B1, C, and F) for visualization and interpretation (Additional file 1: Table S4). The structure of the mu-type opioid receptor (OPRM_HUMAN) belonging to class A was adapted from PDB 8EF6 [44]; the structure of the secretin receptor (SCTR_HUMAN) belonging to class B1 was adapted from PDB 6WZG [45]; the structure of metabotropic glutamate receptor 1 (GRM1_HUMAN) belonging to class C was adapted from PDB 7DGD [46]; the structure of the human smoothened homolog (SMO_HUMAN) belonging to class F was adapted from PDB 6XBM [47].

**Fig. 6:**
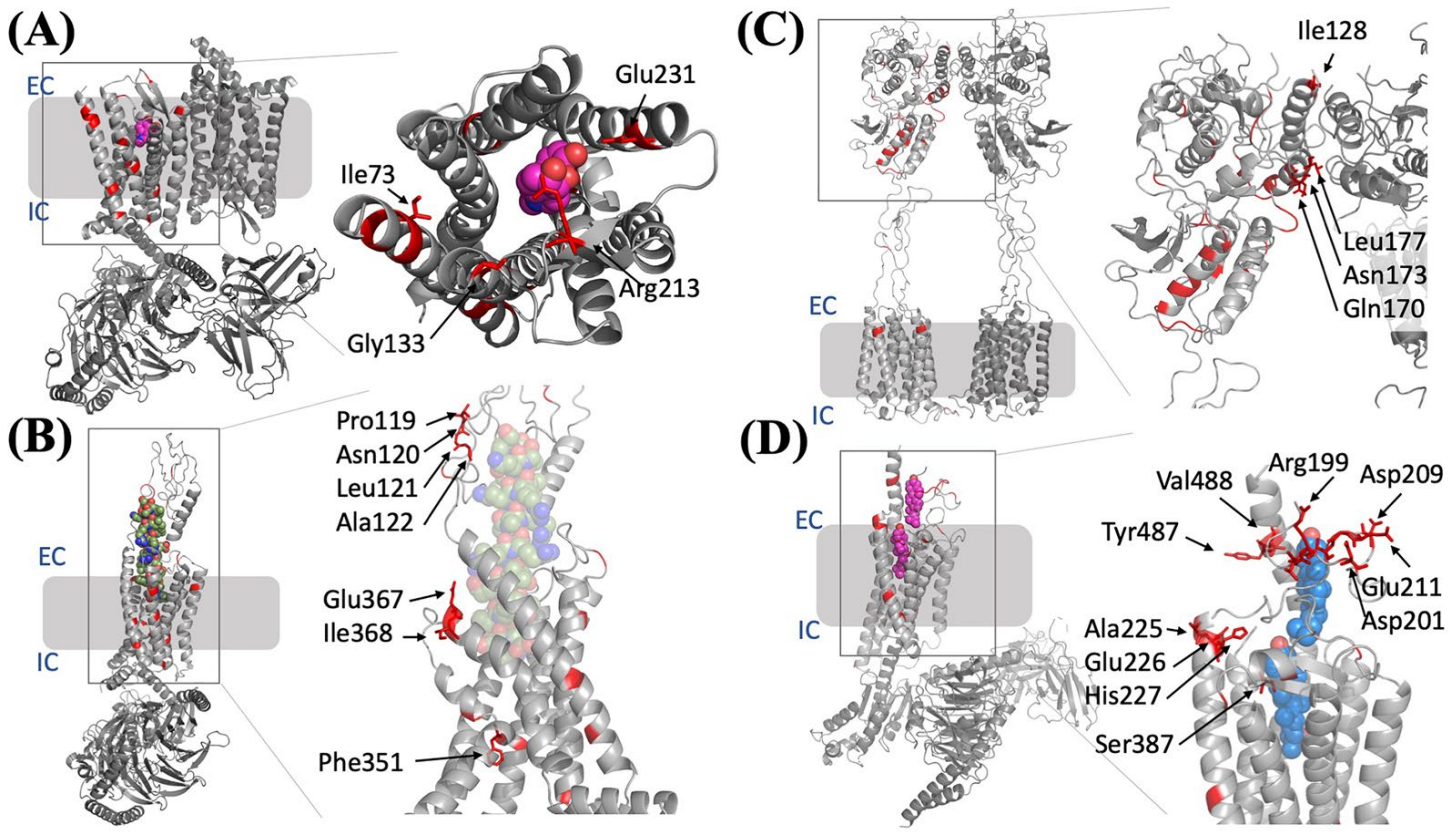
Four GPCR-ligand structures with highlighted top-ranked 200 protein features. **A** Class A GPCR representation protein structure adapted from the mu-type opioid receptor OPRM_HUMAN, PDB 8EF6. The bound-ligand showed as a space-fill format in magenta. **B** Class B1 GPCR representation protein structure adapted from the secretin receptor SCTR_HUMAN, PDB 6WZG. The bound-peptide ligand showed as a space-fill format in dark green. **C** Class C GPCR representation protein structure adapted from the metabotropic glutamate receptor 1 GRM1_HUMAN, PDB 7DGD. **D** Class F GPCR representation protein structure adapted from the human smoothened homolog, SMO_HUMAN, PDB 6XBM. The bound-ligand showed as a space-fill format in dark blue. Abbreviations: EC, extracellular; IC, intracellular

The GPCR-ligand interaction patterns were further analyzed using the selected 162 protein features from the multitask MTL-AG-ATG-FS model and highlighted the associated residues on four adapted protein structures. Knowing that the high diversity of the GPCRs, a significant number of protein features can be associated with gap regions rather than residues. From the class A GPCR structure of the mu-type opioid receptor, 4 out of 17 associated residues (please refer to Additional file 1: Table S4), *i.e.* Ile73, Gly133, Arg213, and Glu231, demonstrated direct ligand contact (Fig. 6A). From the class B1 GPCR structure of the secretin receptor, 19 residues were coherent with the feature-selected residues, and 7 of them showed approaching to its peptide ligand, *i.e.* Pro119, Asn120, Leu121, Ala122, Phe351, Glu367, and Ile368 (Fig. 6B). The class C GPCR structure of metabotropic glutamate receptor 1 including a reported N-terminal extracellular domain showed that the seven transmembrane helices structural scaffolds of the GPCRs are analogous to other classes, and the reported structure additionally highlighted the four selected residues from the MTL-AG-ATG-FS situated in the GPCR-GPCR interaction interface (Fig. 6C). The highlighted residues demonstrated that the region from the N-terminal extracellular domain, in this case, has a higher impact than the seven transmembrane helices in its bioactivities. Furthermore, the class F GPCR structure of human smoothened homolog presented 10 residues out of either direct contact or contributing to the electrostatic interaction, including 7 charging residues, Arg199, Asp201, Asp209, Glu211, Glu226, His227, and Tyr487 (Fig. 6D).

The GPCR crystal structures with ligand-bound form demonstrated the 162 selected protein features covered the protein–ligand interaction residues, and the protein features selected for class A, class B, and class F are predominantly situated on the N-terminal extracellular region and the transmembrane α-helix 1 and 2 (N-TM1-TM2). The features in the N-terminal extracellular domain may involve a dynamic participant in GPCR signaling [42]. The class C GPCR without chemical ligand crystal structures but showing dimer conformation demonstrated that the large N-terminal domain was also mediated in higher-order GPCR-GPCR interaction for its bioactivities [48, 49]. The highlighted residues of four classes were one of the first pieces of evidence that the ligand recognition through the N-terminal region including the extracellular region of the GPCR using the feature-selected multitask model (MTL-AG-ATG-FS), which uptook the knowledge of cross-class protein receptors and learned the knowledge of agonistic and antagonistic ligand information. This method demonstrated a new vision in learning the knowledge of how the enzyme-ligand pair activation within the highly diverse human GPCR superfamily.

## Conclusions

The complexity and diversity of the GPCR family as well as the lack of protein–ligand activity for orphan GPCRs present a difficulty in the development of orphan GPCR-targeted medicines. The present work created multitask models for predicting the EC_50_ values of drug-human GPCR pairs with a special focus on addressing orphan target issues to enable drug development for orphan GPCR. The assessment of several multitask models, including agonist and antagonist models for every GPCR as well as integrated models leveraging all GPCRs, demonstrated that the three integrated models (MTL-AG, MTL-ATG, and MTL-AG-ATG) performed better than the single GPCR models. The integration of agonists and antagonists (MTL-AG-ATG) further improved model performance compared with MTL-AG and MTL-ATG. This indicated that the multitask model was able to inherit the cross-classes GPCR knowledge through the 2,554 aligned protein features. The model was further improved by applying feature selection algorithms to keep only informative features with greater performance and less training time (MTL-AG-ATG-FS). The key protein features were mapped into the 3D GPCR structures and provided insights into the mechanism of the GPCR-ligand interactions.

While the largest database of GPCRdb was utilized in this study, it is still possible to leverage large-scale pre-trained models. Future works could be the use of features generated by using pre-trained models, such as GPT [50], ProtVec [51], MegaMolBART [52], and the adaptation of different multitask feature selection algorithms [53, 54].

This multitask model not only enables the prediction of EC_50_ for the orphan GPCRs, but also provides a new perspective on the combination of proteins and agonistic and antagonistic chemical features to unravel the hidden message of the GPCR superfamily. The developed multitask model enables a more in-depth comprehension of the mechanisms behind the GPCR-ligand interactions and has potential implications in the study of orphan GPCR proteins and the discovery of therapeutic substances. In addition, the transferability of the model to orphan receptors was investigated based on the similarity of protein features. Overall, the proposed method and identified informative residues could facilitate the understanding of the GPCR superfamily and accelerate the development of novel therapeutic substances.

## Scientific contribution

This study has made two significant contributions: (1) introducing the first model for predicting EC_50_ of orphan GPCR-ligand pairs and (2) demonstrating the transferability of data-rich GPCR patterns for orphan GPCR drug discovery. The proposed multitask model based on explainable features is expected to be valuable for GPCR superfamily drug development.

### Supplementary Information

**Additional file 1.** Supplementary tables and figures.

## Data Availability

Data and materials are available on GitHub https://github.com/drhuangwc/GPCR.

## Abbreviations

GPCR: G protein-coupled receptor
CC: Pearson's correlation coefficient
3D: Three-dimension
EC_50_: Half maximal effective concentration
MSE: Mean squared error
MAE: Mean absolute error
FDA: The Food and Drug Administration
RMSD: Root-mean-square deviation
ECFP: Extended-connectivity fingerprints
STL-AG: The single-task learning models for agonist activity of individual GPCRs
STL-ATG: The single-task learning models for antagonist activity of individual GPCRs
MTL-AG: The multitask model for agonist activity of GPCRs
MTL-ATG: The multitask model for antagonist activity of GPCRs
MTL-AG-ATG: The multitask model for a merged of agonist and antagonist activity of GPCRs
MTL-AG-ATG-FS: The multitask model with the top-ranked 200 feature-selected for a merged of agonist and antagonist activity of GPCRs
mRMR: The minimum redundancy and maximum relevance algorithm
Ts: Tanimoto similarity
PDB: Protein data bank
TM1: Transmembrane α-helix 1
TM2: Transmembrane α-helix 2
EC: Extracellular
IC: Intracellular
