# Supplementary material for: Decrypting orphan GPCR drug discovery via multitask learning
Source: J Cheminform. 2024 Jan 23;16:10. doi: 10.1186/s13321-024-00806-3 (PMC10804799; doi:10.1186/s13321-024-00806-3)
Supplement: Supplementary file 1 — Additional file 1. Supplementary tables and figures. [file 13321_2024_806_MOESM1_ESM.docx]

**Decrypting Orphan GPCR Drug Discovery via Multitask Learning**

**Wei-Cheng Huang^1^, Wei-Ting Lin^1^, Ming-Shiu Hung^1^, Jinq-Chyi Lee^1^, Chun-Wei Tung^1*^**

^1^ Institute of Biotechnology and Pharmaceutical Research, National Health Research Institutes, Miaoli County, Taiwan

* Correspondence: [cwtung@nhri.edu.tw](mailto:cwtung@nhri.edu.tw)

**Supplementary Information**

Table S1. The validation performance of the single-task and multitask models. Pearson's correlation coefficient (CC), mean squared error (MSE), and mean absolute error (MAE) were used to evaluate the models.

| **Models** | **Evaluation using validation dataset** | | | | | | **Evaluation using orphan dataset** | |
| --- | --- | --- | --- | --- | --- | --- | --- | --- |
|  | ***AutoML model*** | ***Number of models*** | ***Model Training Time*** | ***MSE*** | ***CC*** | ***MAE*** | ***MSE*** | |
|  |  |  |  |  |  |  | ***Number of GPCRs*** | **Mean *(SEM)*** |
| STL-AG | - | *150* | - | 0.96 | 0.83 | 0.46 |  |  |
| MTL-AG | WeightedEnsemble_L3 | *1* | 3.5 hrs | 0.29 | 0.80 | 0.37 |  |  |
| STL-ATG | - | *155* | - | 0.50 | 0.83 | 0.47 |  |  |
| MTL-ATG | WeightedEnsemble_L3 | *1* | 3.5 hrs | 0.27 | 0.83 | 0.35 |  |  |
| MTL-AG-ATG | WeightedEnsemble_L3 | *1* | 7 hrs | 0.24 | 0.85 | 0.30 | *16* | 1.7 |
| MTL-AG-ATG-FS | WeightedEnsemble_L3 | *1* | 1.7 hrs | 0.24 | 0.85 | 0.30 | *16* | 1.5 |
| MTL-AG-ATG-M2V | WeightedEnsemble_L3 | *1* | 7 hrs | 0.27 | 0.85 | 0.33 | *16* | 1.7 |
| MTL-AG-ATG-M2V-FS | WeightedEnsemble_L3 | *1* | 1.7 hrs | 0.27 | 0.84 | 0.33 | *16* | 1.5 |

Table S2. Summary of feature selection results. Pearson's correlation coefficient (CC) and mean squared error (MSE) were used to evaluate model performance. *The final feature set for training the MTL-AG-ATG-FS model.

| **Number of Top-Ranked**  **Features**  **(*m*)** | **Number of Protein Features** | **Number of Physicochemical Features** | **Total Number of Features (n)** | **Training Time (min)** | **MSE** | **CC** |
| --- | --- | --- | --- | --- | --- | --- |
| 1 | 1 | 0 | 1025 | 85.5 | 0.431 | 0.891 |
| 2 | 1 | 1 | 1026 | 85.7 | 0.431 | 0.891 |
| 5 | 3 | 2 | 1029 | 85.8 | 0.363 | 0.909 |
| 10 | 7 | 3 | 1034 | 86.3 | 0.316 | 0.922 |
| 15 | 11 | 4 | 1039 | 86.7 | 0.296 | 0.927 |
| 20 | 15 | 5 | 1044 | 87.2 | 0.290 | 0.928 |
| 25 | 19 | 6 | 1049 | 87.5 | 0.289 | 0.929 |
| 30 | 24 | 6 | 1054 | 88.0 | 0.289 | 0.929 |
| 50 | 41 | 9 | 1074 | 89.7 | 0.270 | 0.934 |
| 100 | 82 | 18 | 1124 | 93.8 | 0.254 | 0.938 |
| 200* | 162 | 38 | 1224 | 102.2 | 0.249 | 0.939 |
| 300 | 242 | 58 | 1324 | 110.5 | 0.249 | 0.939 |
| 500 | 371 | 127 | 1522 | 127.0 | 0.247 | 0.939 |
| 1000 | 628 | 345 | 1997 | 166.5 | 0.245 | 0.940 |
| 1500 | 812 | 545 | 2381 | 198.5 | 0.245 | 0.940 |
| 2000 | 947 | 749 | 2720 | 226.8 | 0.245 | 0.940 |
| 2500 | 1097 | 903 | 3024 | 252.2 | 0.245 | 0.940 |
| 3000 | 1305 | 1009 | 3338 | 278.3 | 0.248 | 0.939 |
| 3500 | 1555 | 1117 | 3696 | 308.2 | 0.248 | 0.939 |
| 4500 | 2255 | 1244 | 4523 | 377.0 | 0.248 | 0.939 |
| All | 2554 | 1444 | 5022 | 418.7 | 0.248 | 0.939 |

Table S3. Informative protein features and physicochemical features of ligands for the MTL-AG-ATG-FS model

|  | Informative Features of the MTL-AG-ATG-FS model |
| --- | --- |
| 162 Protein Features | **1^st^ part:** [0, 1, 2, 3, 4, 5, 6, 7, 8, 9, 10, 11, 12, 16, 24, 25, 31, 37, 41, 45, 57, 62, 64, 69, 79, 94, 101, 104, 108, 152, 153, 155, 156, 157, 158, 159, 241, 271, 272, 273, 274, 275, 276, 277, 278, 279, 280, 281, 282, 283, 284, 285, 286, 287, 288, 289, 290, 291, 308, 309, 310, 311, 312, 313, 349, 350, 351, 352, 353, 354, 355, 356, 359],  **2^ed^ part:** [422, 450, 451, 453, 458, 459, 460, 462, 463, 464, 465, 466, 467, 468, 470, 471, 472, 473, 474, 476, 477, 478, 479, 480, 483, 484, 485, 486, 487, 488, 489, 490, 492, 493, 495, 499, 505, 507, 508, 518, 519, 520, 522, 531, 532, 533, 535, 536, 537, 538, 539, 540, 542, 543, 544, 545, 546, 547, 549, 554, 555, 556, 557, 560, 562, 563, 564, 594, 595, 596, 597, 640, 652, 686],  **3^rd^ part:** [752, 759, 807, 951],  **4^th^ part:** [1136, 1359],  **5^th^ part:** [1663, 1664, 1748, 1750, 1793, 1794],  **6^th^ part:** [1904],  **7^th^ part:** [2341, 2344] |
| 38 Physicochemical Features | **Autocorrelation:**  51 ATS2p, 132 ATSC2c, 174 ATSC8p,  **Burden modified eigenvalues:**  487 SpMin3_Bhm, 488 SpMin4_Bhm, 490 SpMin6_Bhm, 491 SpMin7_Bhm, 503 SpMin3_Bhv, 521 SpMin5_Bhe, 522 SpMin6_Bhe,  568 SpMin4_Bhs,  **Chi path:**  611 SP-5, 612 SP-6, 625 VP-3, 626 VP-4, 627 VP-5,  **Detour matrix:**  655 SpMAD_Dt,  **Atom type electrotopological state:**  1104 maxsSH, 1143 gmax,  **Extended topochemical atom:** 1182 ETA_Eta,  **Hbond donor count:**  1203 nHBDon_Lipinski,  **Information content:**  1206 IC1, 1207 IC2, 1208 IC3, 1209 IC4, 1222 SIC5,  **Path counts:**  1282 MPC4,  **Ring count:** 1357 nF11HeteroRing,  **Topological distance matrix:**  1403 SpMAD_D,  **Van der Waals volume:**  1412 VABC,  **Vertex adjacency information (magnitude):** 1413 VAdjMat,  **Walk counts:**  1414 MWC2, 1415 MWC3, 1416 MWC4, 1423 TWC, 1425 SRW3, 1427 SRW5, 1429 SRW7 |

Table S4. The informative residues of four human GPCRs mapped from the MTL-AG-ATG-FS model.

| Class A  OPRM_HUMAN | A18, P47, I71, T72, I73, M74, F89, I95, A104, L118, L123, G133, T162, R213, E231, F291, V293 |
| --- | --- |
| Class B1  SCTR_HUMAN | M1, E67, S98, P119, N120, L121, A122, L154, A160, T173, L187, I194, A210, V235, A274, F351, I353, E367, I368 |
| Class C  GRM1_HUMAN | F7, F8, P10, A11, I12, F13, L14, L96, I128, L155, L156, Q157, L158, I161, P162, Q163, I164, A165, Y166, S167, A168, S170, I171, L173, T177, L183, V185, V186, M196, L197, D198, V200, T206, Y207, V208, A210, G215, N216, Y217, G218, G221, D223, A224, F225, L262, F271, V289, T320, P326, R345, C420, N571, G646, L647, S692, L694, L725, I726 |
| Class F  SMO_HUMAN | G40, P41, S43, A44, G45, G46, S47, A87, G104, K105, L106, V107, L108, W109, S110, G111, N114, V157, E176, G177, N184, K186, F187, L197, V198, R199, D201, D209, E211, G212, C213, A225, E226, H227, L243, L249, L267, M286, I293, G304, V334, S387 |

Figure S1. The performance under various similarity thresholds on the orphan datasets of the (A) MTL-AG-ATG model and (B) MTL-AG-ATG-FS model.


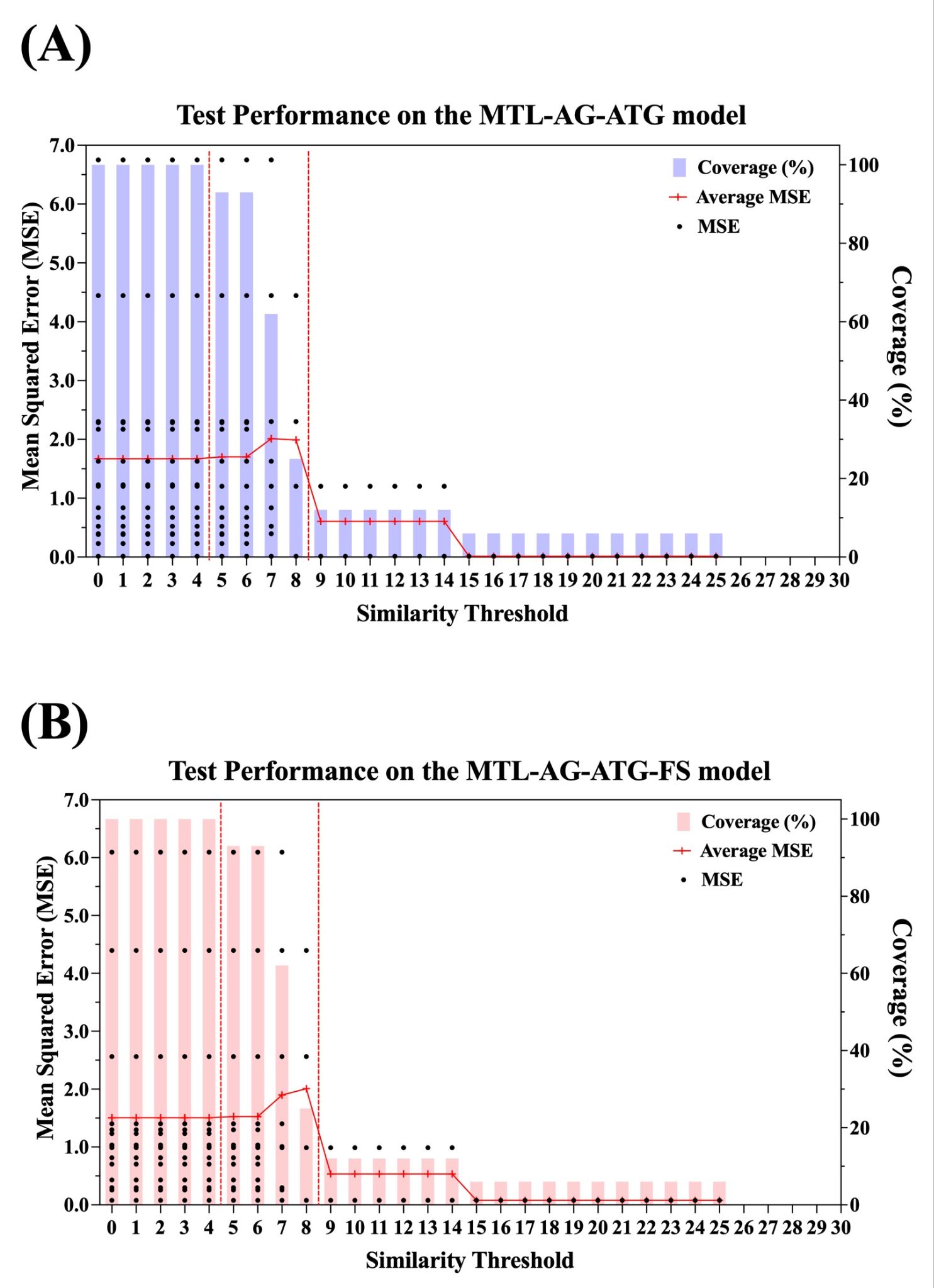


Table S5. The table of predicted EC_50_ values using the MTL-AG-ATG-FS model for the reported agonistic ligands of GPRC5A [1].

| 7-Fluorotryptamine (7FTA) 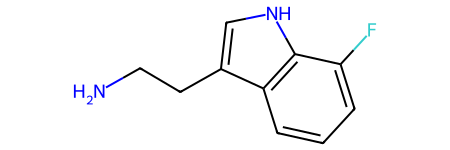 | Tryptamine (TA)  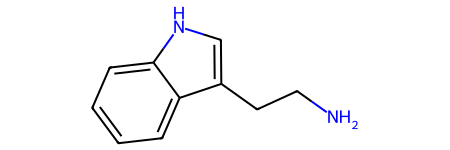 |
| --- | --- |
| C1=CC2=C(C(=C1)F)NC=C2CCN | C1=CC=C2C(=C1)C(=CN2)CCN |
| Predicted LogEC_50_: 3.4 | Predicted LogEC_50_: 3.3 |
| Experimental LogEC_50_: 3.9 | Experimental LogEC_50_: 4.9 |

**References**

1. Zhao X, Stein KR, Chen V, Griffin ME, Lairson LL, Hang HC (2023) Chemoproteomics reveals microbiota-derived aromatic monoamine agonists for GPRC5A. Nat Chem Biol 19:1205-1214. doi:10.1038/s41589-023-01328-z
